# *In vitro* modeling of hepatocellular carcinoma niche on decellularized tomato thorny leaves: a novel natural three-dimensional (3D) scaffold for liver cancer therapeutics

**DOI:** 10.3389/fbioe.2023.1189726

**Published:** 2023-05-05

**Authors:** Mariye Ahmadian, Saadi Hosseini, Atefeh Alipour, Mehdi Jahanfar, Naser Farrokhi, Shahin Homaeigohar, Hosein Shahsavarani

**Affiliations:** ^1^ Department of Cell and Molecular Biology, Faculty of Life Science and Biotechnology, Shahid Beheshti University, Tehran, Iran; ^2^ Laboratory of Regenerative Medicine and Biomedical Innovations, Pasteur Institute of Iran, National Cell Bank, Tehran, Iran; ^3^ Department of Nanobiotechnology, Pasteur Institute of Iran, Tehran, Iran; ^4^ School of Science and Engineering, University of Dundee, Dundee, United Kingdom

**Keywords:** liver cancer modeling, tumor microenvironment, 3D culture, natural cellulose scaffolds, drug screening

## Abstract

Liver cancer is now one of the main causes leading to death worldwide. To achieve reliable therapeutic effects, it is crucial to develop efficient approaches to test novel anticancer drugs. Considering the significant contribution of tumor microenvironment to cell’s response to medications, *in vitro* 3D bioinspiration of cancer cell niches can be regarded as an advanced strategy to improve the accuracy and reliability of the drug-based treatment. In this regard, decellularized plant tissues can perform as suitable 3D scaffolds for mammalian cell culture to create a near-to-real condition to test drug efficacy. Here, we developed a novel 3D natural scaffold made from decellularized tomato hairy leaves (hereafter called as DTL) to mimic the microenvironment of human hepatocellular carcinoma (HCC) for pharmaceutical purposes. The surface hydrophilicity, mechanical properties, and topography measurement and molecular analyses revealed that the 3D DTL scaffold is an ideal candidate for liver cancer modeling. The cells exhibited a higher growth and proliferation rate within the DTL scaffold, as verified by quantifying the expression of related genes, DAPI staining, and SEM imaging of the cells. Moreover, prilocaine, an anticancer drug, showed a higher effectiveness against the cancer cells cultured on the 3D DTL scaffold, compared to a 2D platform. Taken together, this new cellulosic 3D scaffold can be confidently proposed for chemotherapeutic testing of drugs on hepatocellular carcinoma.

## 1 Introduction

Liver cancer is one of the most prevalent types of cancer that is ranked fourth among the main causes of cancer-related deaths across the world. Liver cancer was predicted to be the cause of 830,000 deaths in 2020 (Sung et al., 2021). Considerable efforts have been carried out towards the development of efficient cancer treatments. In this regard, discovery, and validation of new, effective anticancer drugs for both chemotherapy and daily medication have been diligently pursued. Cell-based assays are used in the investigations to gain insights into cellular responses to various drugs in an inexpensive, time-effective, and scalable manner (Lovitt et al., 2014). A high demand for developing innovative, reliable, and consistent models for drug testing and hepatotoxicity research is existent (Hughes, 2008). The employment of tissue engineering technologies for cancer biology has been appealing, as they provide a suitable milieu that can regulate tumor development and progression, and metastasis thereof. Tumor cells communicate with their microenvironment in a very dynamic and reciprocal way. Therefore, implementation of custom-designed biomaterial scaffolds instead of conventional monolayer tissue culture models can promise more reliable therapeutic feedback (Liu and Vunjak-Novakovic, 2016). The proper design of cell culture conditions for cancer research is crucial to achieve better understanding of tumor biology. This will lead to the discovery of optimum and efficient radio/chemotherapy parameters and the development of state of the art cancer therapeutic strategies (Aggarwal et al., 2009).

In most of studies, tumor cells have been cultured on 2D surfaces, where cell-cell interactions, cell behavior and responses to external stimuli are far from reality (Skardal et al., 2012; Antoni et al., 2015). Thus, model structures that better represent what is happening in the actual tissues, while analyzing chemosensitivity of carcinoma cells to different anti-tumor medicines, are highly demanded. The 3D cell culture platforms have been designed to properly simulate *in vivo* conditions and thus develop a microenvironment that is nearly identical to the native one. This allows cell growth in all three dimensions and enables the cells to interact with one another and with the components of the surrounding matrix, as they do in the tissue microenvironment (Lovitt et al., 2014; Rijal and Li, 2016).

The human hepatocarcinoma (HepG2) cell line is frequently utilized for modelling of liver cancer cells for the purpose of drug screening. It is believed that proliferation of HepG2 cells in ideal 3D cell cultures will promote tumor-like functionality, thus providing reliable data (Guo et al., 2011; Aritomi et al., 2014).

Despite substantial advances in the creation of tissue engineering scaffolds, nutrients delivery to intricately engineered human tissues is still challenging (Gershlak et al., 2017). Materials of natural origin are among the safest and easiest to obtain biomedical tools among the diverse possible sources. In the recent years, tissue engineering researchers have become more interested in the development of nature-derived scaffolds with plant and animal tissue origins (Predeina et al., 2020; Mostofi et al., 2022). A plant tissue can be decellularized by chemical methods and the remining cellulose skeleton can act as a scaffold for mammalian cell culture. The decellularized plant tissue is in fact a promising 3D scaffold superior to the synthetic polymer nonwovens or the scaffolds that can be obtained from animals with reported side effects (Aghazadeh et al., 2022).

Due to biocompatibility, permeability, and eco-friendliness, various cellulose scaffolds can support a large variety of mammalian cells and therefore are suitable to study cell-drug responses (Luo et al., 2019; Salehi et al., 2020a; Salehi et al., 2020b; Phan et al., 2020; Aghazadeh et al., 2022). For instance, the research on cancer cells describes the possibility of mechano-regulation of the prostate cancer (PC3) cells and the melanoma (SK-MEL-28) cells cultured on the scaffolds made from decellularized spinach leaf in contrast to the cells cultured on a rigid conventional cell culture platform. These cells were shown to have proper sensitivity to radiation and drug treatment as well. According to this research, in comparison to standard cell culture models, plant decellularization provides soft scaffolds with stiffness comparable to majority of human tissues’ and improve cell behavior, particularly when they are exposed to radiation and drug (Lacombe et al., 2020). The decellularized scaffolds are biocompatible, naturally perfused, adjustable, and easy to prepare, and can be employed as eco-friendly tissue engineering materials (Bilirgen et al., 2021).

In this study, we decellularized a plant tissue to fabricate a 3D pre-vascularized scaffold for tissue engineering by considering the resemblance between the vascular network of animal and plant tissues. A common HCC derived cell line, i.e., HepG2, was used to culture on the 3D DTL scaffold featuring tiny surface trichomes that could mimic the microenvironment of liver cancer cells. Additionally, the efficacy of prilocaine on the cells cultured on the DTL scaffold was compared with that on the cells cultured on a 2D tissue culture plate.

## 2 Materials and methods

### 2.1 Sample collection

The leaves of tomato plant (*Solanum lycopersicum* L.) were obtained from a local greenhouse in Tehran, Iran. The fresh leaves were quickly decellularized, freeze-dried, and stored at 4°C.

### 2.2 Materials

Sodium dodecyl sulfate (SDS), 3-(4,5-dimethyltiazole-2-yl)- 2,5-diphenyltetrazolium bromide (MTT), dimethyl sulfoxide (DMSO), 4′,6-diamidino-2-phenylindole (DAPI), Acridine Orange/Propidium Iodide and DMEM/Ham’s F12 medium were obtained from Sigma–Aldrich (Burlington, VT, United States). Fetal bovine serum (FBS) and Pen-Strep solution were purchased from Gibco (New York, NY, United States). Phosphate-buffered saline (PBS) was purchased from FUJIFILM Wako Pure Chemical Corporation (Osaka, Japan). Prilocaine (3%), a local anesthetic drug, was obtained from Exir Pharmaceutical Company, Iran. Alkaline phosphatase kit was bought from Pars Azmoon (Tehran, Iran). DENAzist Column RNA Isolation Kit was obtained from DENAzist Asia Co., (Mashhad, Iran). cDNA synthesis kit was purchased from Parstous Co. (Tehran, Iran).

### 2.3 Scaffold preparation

The decellularization process was carried out according to a previously described method (Salehi et al., 2021). In brief, tomato leaves were soaked twice in deionized water (DW) for 5 min and then rinsed in PBS three times to completely remove wax coating from the surface of the leaves. The leaves were immersed in SDS (10%) at 25°C for 5 days, washed three times in DW and eventually incubated in sodium hypochlorite solution (10%) for 3 h. The leaves were thoroughly washed in distilled water, hexane (98%), and lastly in 1× PBS for 1 min each.

The scaffolds were freeze-dried for preservation purpose. The decellularized leaves were frozen in liquid N_2_ and placed in a freeze dryer (−50°C, Christ Alpha 1-2, Germany) for 5 h. The scaffolds were stored within a fridge at 4°C. To increase the hydrophilicity of the scaffolds and thus to improve cell adhesion, the scaffolds were exposed to plasma for 1 min within an expanded tabletop plasma cleaner (AC/DC input 230 V, AC (PDC-002/PDC-FMG 2) of 150 W).

**FIGURE 1:**
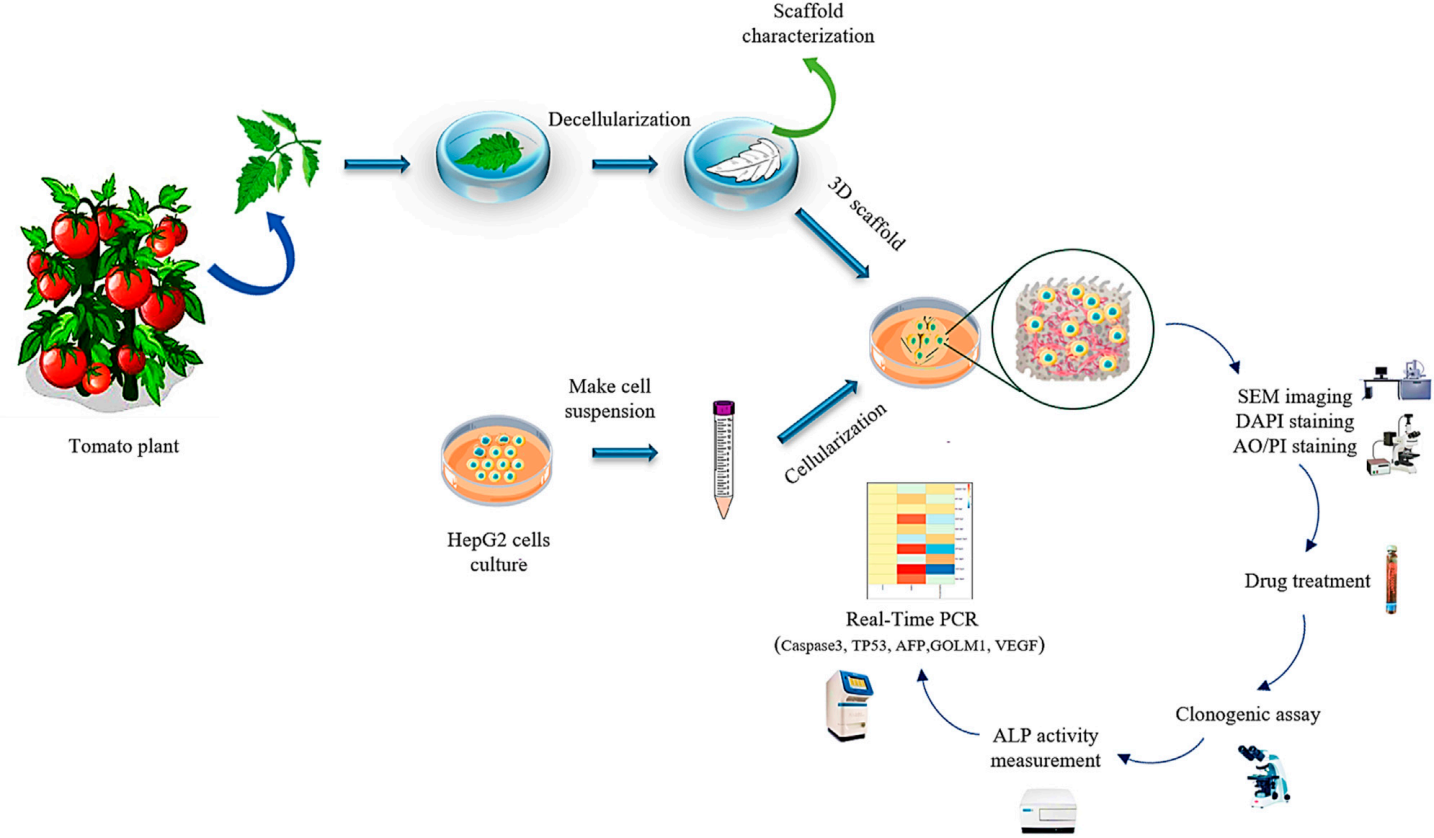
The schematic illustration of the preparation process of the decellularized tomato thorny leaf (DTL) scaffold and characterization of HepG2 cells behavior thereon.

To ascertain about decellularization of the plant tissue, the DNA content of fresh and decellularized tomato leaves were compared. For this purpose, 20 mg dry weight of the plant tissues before and after decellularization were ground under liquid N_2_ to a fine powder and transferred into a new microcentrifuge tube. The DNA content of the samples (fresh and decellularized) was determined using FAVORGEN DNA extraction kit, while their concentration was measured by a UV-Vis spectrophotometer (Victor 3, PerkinElmer, Waltham, MA) at the wavelength of 260 nm. The experimental procedure of this study is schematically shown in Figure 1.

### 2.4 Scaffold characterization

Scanning electron microscopy (SEM): The morphological characteristics of the decellularized scaffolds were assessed via scanning electron microscopy (FEI SEM QUANTA 200, United States). To do so, the freeze-dried samples were sputter coated with a thin Au layer (10 nm).

Fourier Transform Infrared (FTIR): Surface chemical composition of the DTL scaffold was determined using ATR-FTIR (Thermo Nicolet Avatar 380). The samples were exposed to IR radiation at 450–4,000 cm^–1^ spectral region (22°C, 20% relative humidity).

Brunauer–Emmett–Teller (BET) analysis: The pore diameter, pore volume, and surface area of the scaffolds were quantified using a BET instrument (BELSORP MINI II). The scaffolds were put within a degassing machine (BEL PREP VAC II) for 2 h at 120°C, and nitrogen adsorption isotherms were obtained at the bath temperature range of −196°C to 15°C.

Contact angle measurement: The hydrophilicity of the plasma treated and the pristine DTL scaffold was quantified by a JIKAN CAG-20 device after deposition of water droplets on four locations of the scaffold surface (1 cm^2^).

Swelling behavior: The scaffolds' swelling behavior was determined following a previously established protocol (Katt et al., 2016; Saydé et al., 2021). After freeze-drying, the scaffolds' primary weight (W_0_) was measured. The scaffolds were incubated in 1× PBS (pH 7.4°C and 37°C) and weighed after different intervals (1, 2, 4, 8, 16, and 24 h) (W_t_). The swelling percentage of the scaffolds was determined via Equation 1:

$$Swelling\;\%=\frac{\left(W_{t}-W_{0}\right)}{W_{0}}\times 100$$ (1)

Degradation behavior: The degradation percentage of the scaffolds was measured as below (Esmaeili et al., 2022): the primary weight of the freeze-dried scaffolds (W_0_) was determined. The scaffolds were then incubated in 1× PBS at pH7.4 at 37°C for 10, 20, 30, 60, and 90 days. The scaffolds were removed from PBS, repeatedly washed with distilled water, freeze-dried, and eventually weighed (W_t_). The scaffold’s degradation percentage was calculated via Eq. 2:

$$Degradation\;\%=\frac{\left(W_{0}-W_{t}\right)}{W_{0}}\times 100$$ (2)

Cytotoxicity: Cytotoxicity and cell viability of the scaffolds were characterized through a MTT assay. To do so, human foreskin fibroblasts (HFFs) and HepG2 cells (both from National Cell Bank, Pasteur Institute of Iran, Tehran, Iran) were co-cultured with the scaffolds that were previously sterilized by UV light irradiation followed by an ethanol (70% v/v) treatment. After incubation for 1, 3, 5, and 7 days, the cell culture medium was removed, and 10 µL of a MTT solution (50 mg MTT in 10 mL PBS) was poured into each scaffold containing well. Each well was filled with 90 µL of culture medium and incubated for 4 h. Afterwards, the culture medium and MTT were aspirated out. 100 μL DMSO was added to the culture medium to dissolve the formed Formazan crystals. The well-plates were eventually incubated within a shaking incubator (for 20–30 min at 37°C). The optical absorbance of the medium was read by an ELISA reader (Bio-Rad, Hercules, CA, United States) at the wavelength of 590 nm.

### 2.5 Cell culture

After decellularization and freeze-drying, the DTL scaffolds were sterilized by UV light irradiation for 40 min and then incubation in ethanol (70% v/v) for 2 min. The scaffolds were washed three times with 1× PBS and penicillin/streptomycin (2.5% w/v) for 15 min. The samples were placed into several wells of a (24-well) cell culture plate filled with culture medium and then incubated in 5% CO_2_, 90% humidity, and at 37°C for 12 h. The HepG2 cells were seeded on tissue culture plate (TCP) and the decellularized scaffolds with the cell density of 5 × 10^4^ cell/well. Afterwards, 500 µL DMEM/Ham’s F12 medium supplemented with FBS (10%), sodium bicarbonate (15 mM), penicillin/streptomycin (1% w/v), and glutamate (2 mM) were poured into the wells. Eventually, the cell-scaffold co-cultures were incubated under 5% CO_2_ at 37°C.

### 2.6 Prilocaine treatment

To evaluate the applicability of the DTL scaffolds in drug screening, the effect of prilocaine on the liver cancer cells seeded on the DTL scaffolds was investigated. The half maximal inhibitory concentration (IC_50_) for normal cells was determined via a MTT assay. For this purpose, we used prilocaine 3% (30 mg/mL) and treated the cells with different concentrations of prilocaine (including 1/2,1/4,1/8,1/16, 1/32, 1/64, 1/128, 1/256, 1/512, 1/1024, and 1/2048). The optical absorbance of the medium was read by an ELISA reader (Bio-Rad, Hercules, CA, United States) at the wavelength of 590 nm 72 h after the treatment and the optimum concentration was identified. The cells cultured on TCP (2D substrate) and on the DTL scaffold were exposed to the drug on the 5th and 11th days of the incubation period. Any alterations in gene expression by the cells and in the number of the cell colonies formed on the 2D and 3D substrates after the drug treatment were assessed on the 7th and 14th days of the cell culture (after 48 and 72 h).

### 2.7 Cell adhesion on the DTL scaffold

#### 2.7.1 DAPI staining

The cell nuclei were stained using DAPI to examine the cell adhesion and the cell viability on the DTL scaffold. After 7 incubation days, the cells were fixed using paraformaldehyde (4%) for 0.5 h, washed with 1× PBS three times, and subsequently treated with 5 μg/mL DAPI reagent for 10 min. The DTL scaffold was washed two times with 1× PBS, and then was imaged by a fluorescence microscope [Bell Engineering INV100-FL, Monza (MB), Italy].

#### 2.7.2 Scanning electron microscopy (SEM)

The growth, attachment, and expansion of HepG2 cells on the DTL scaffold were visualized by SEM on the 14th incubation day. The HepG2 cells were fixed on the DTL scaffold with glutaraldehyde/PBS solution (2.5%) for 16 h at 4°C and later washed with 1× PBS three times. The samples were subsequently dehydrated in ethanol (increasing concentration of 20%–96% v/v) for 15 min each and left to dry. The scaffold was gold-coated using COXEM ion-sputtering device and imaged on a FEI SEM QUANTA 200.

### 2.8 Cell proliferation and cell viability

Acridine Orange (AO)/Propidium Iodide (PI) staining was used to image the cell proliferation and the number of dead and live cells on the DTL scaffold. The staining reagents were combined (50 μg/mL (AO) and 10 μL (PI)) and added to the cell culture medium on the 5th incubation day. The cell viability was examined using a fluorescent microscope [Bell Engineering INV100-FL, Monza (MB), Italy].

### 2.9 Clonogenic assay

The cells were cultured on the 3D DTL scaffold and on 2D TCP for 7 days, while part of them were treated with prilocaine on the 5^th^ day. The HepG2 cells were fixed with glutaraldehyde (2.5% v/v) and then stained with crystal violet (0.5% v/v). The colonies containing over 50 cells were counted, and the changes in the number of cell colonies (treated with and without prilocaine) on the 2D and 3D substrates were compared*.*

### 2.10 Alkaline phosphatase (ALP) activity

After 7 incubation days, ALP activity of the cells on the DTL scaffold before and after the drug treatment was assessed using an ALP kit according to the manufacturer’s instruction. The optical density of the cell samples was quantified at the wavelength of 405 nm using a microplate reader (BioTek Epoch, Santa Clara, CA, United States).

### 2.11 Real-Time PCR

Real-Time PCR (StepOne ABI Real-Time PCR system) was employed to quantify the relative expression of HepG2 genes including *Vascular Endothelial Growth Factor (VEGF), Alpha Fetoprotein (AFP), Cysteinyl Aspartate Proteases 3(Caspase-3), Golgi Membrane Protein 1 (GOLM1), Tumor Protein 53 (TP53)*, as well as *GAPDH* reference gene. CLCwork and Oligo7 software were used to design the primer sequences (Table 1).

**TABLE 1:** The primer sequence of HepG2 genes.

Genes	Forward primer sequence (5´→ 3´)	Reverse primer sequence (5´→ 3´)	Product length (bp)
AFP	ACG​GAC​ATT​CAG​ACT​GCT​GC	TGG​AGT​GGG​CTT​TTT​GTG​TGC	77
VEGF	GTA​CCT​CCA​CCA​TGC​CAA​GT	AAT​AGC​TGC​GCT​GGT​AGA​CG	107
Caspase 3	GAA​GCG​AAT​CAA​TGG​ACT​CTG​G	GCA​TCG​ACA​TCT​GTA​CCA​GAC	146
GOLM1	GGA​GCC​TCG​AAA​AGA​GAT​TCT​CAG	GCC​TTC​CAG​CTC​CAT​GAT​CC	190
TP53	AGG​TTG​GCT​CTG​ACT​GTA​CC	GAT​TCT​CTT​CCT​CTG​TGC​GC	195
GAPDH	GTC​TCC​TCT​GAC​TTC​AAC​AGC​G	CAC​CCT​GTT​GCT​GTA​GCC​AA	168

Total RNA content was isolated on the 7th and 14th days of cell culture on the DTL scaffold with and without drug by a DENAzist Column RNA Isolation Kit. Electrophoresis (1.8% agarose gel) and NanoDrop (Agilent, Milan, Italy) were used to evaluate the quality and the quantity of RNA, respectively. Using cDNA synthesis kit (according to the manufacturer’s instructions), single-strand cDNA was synthesized. Based on the comparative Ct method (∆∆Ct) with normalization to GAPDH (Salehi et al., 2020b), gene expression changes were monitored. The GraphPad Prism 8 software and the relative expression software tool (REST) were eventually employed to analyze the collected data.

### 2.12 Statistical analysis

All the tests were carried out three times. The obtained data were presented as mean ± SD. GraphPad Prism 8 was employed for data analysis via two-way analysis of variance (ANOVA) including *t*-test. The *p*-values< 0.05 were regarded as significant.

## 3 Results

### 3.1 Physicochemical characteristics of the DTL scaffold

**FIGURE 2:**
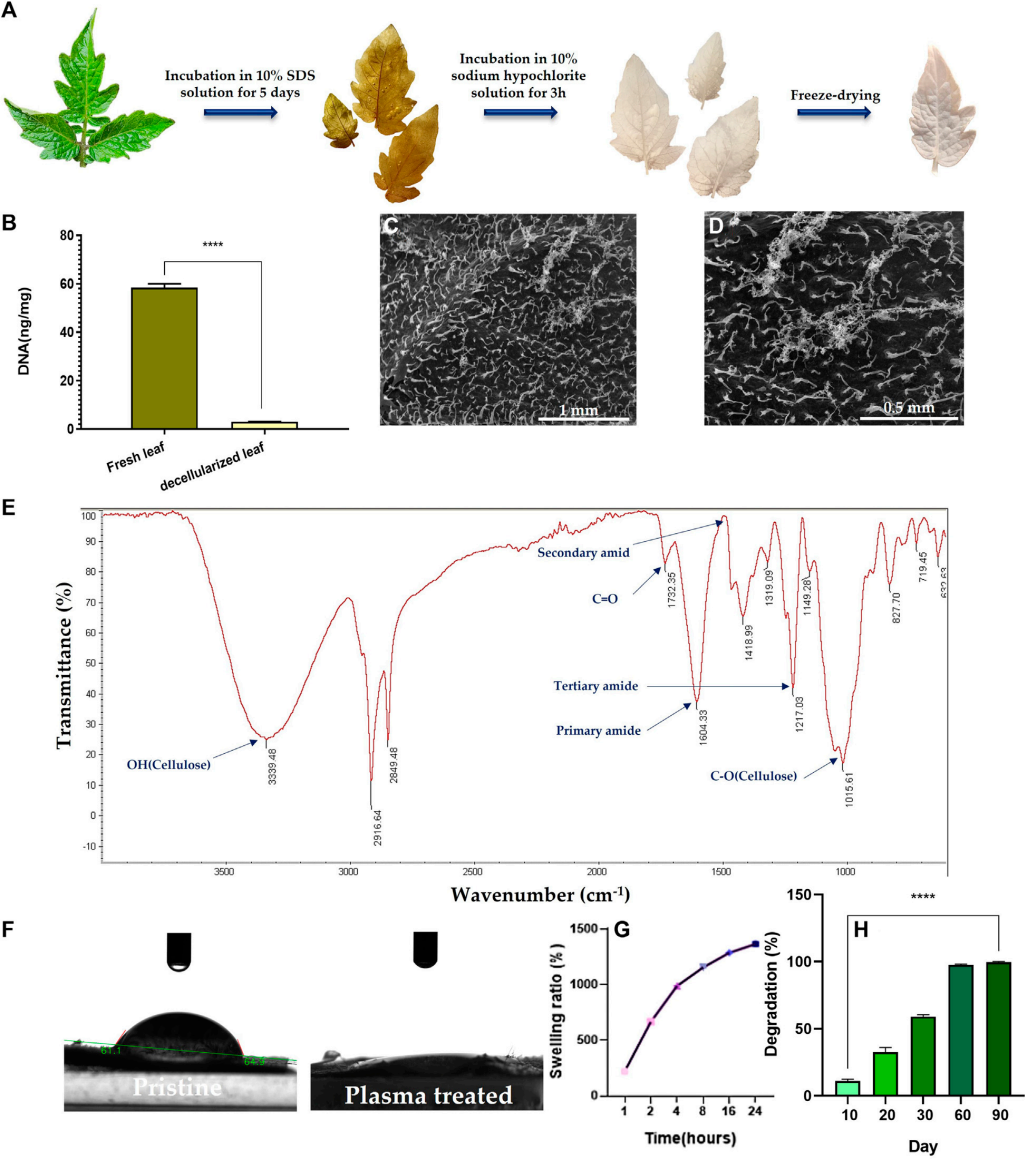
**(A)** Camera images demonstrate color evolution of tomato leaves over the course of the decellularization process. **(B)** DNA content of the decellularized tomato leaf compared to the fresh (pristine) leaf (****: *p* < 0.0001). **(C, D)** SEM images of the surface of the decellularized tomato leaf at two different magnifications (100x **(C)** & 200x **(D)**). **(E)** ATR-FTIR spectrum of the decellularized tomato leaf. **(F)** Camera images visualize the water contact angel on (hydrophilicity of) the decellularized tomato leaf before and after the plasma treatment. **(G)** Swelling kinetics of the decellularized tomato leaf immersed in PBS over 24 h **(H)** Degradation kinetics of the decellularized tomato leaves immersed in PBS over a 90-day incubation period.

The decellularization process was monitored with naked eye. As seen in Figure 2A, green fresh tomato leaves turn white after decellularization, implying the success of the treatment, as similarly verified for decellularization of grass blades by Allan et al. (Allan et al., 2021). The DNA content of the decellularized and the pristine scaffolds was also recorded to confirm the efficiency of the decellularization process. As shown in Figure 2B, the DNA content in the tomato leaves notably declines from 59 ng DNA/mg tissue before decellularization to 3 ng DNA/mg tissue for the decellularized leaves. This means that 95% of the plant DNA is removed by the SDS treatment of the tomato leaves.

As shown in SEM images (Figure 2C), the surface of the tomato leaves features tiny dense trichomes, which partially remain even after decellularization (Figure 2D). ATR-FTIR analysis of the decellularized tomato leaves implies the co-existence of OH, amide, and C-O groups that are associated to cellulose (Figure 2E). Moreover, the appearance of carbonyl bond (C=O) represents ketone. The availability of amide dips at 1604 cm^-1^ (primary) and 1217 cm^-1^ (tertiary) indicates the persistent existence of plant proteins that are not released during the decellularization process.

After the plasma treatment, surface hydrophilicity of the DTL scaffold rises, as shown in Figure 2F. While the water contact angle of the DTL scaffold was measured to be 61°, it majorly dropped to almost zero after the plasma treatment, implying the creation of a supportive substrate for cell adhesion. It is known that surface hydrophilicity can affect protein adsorption and cell adhesion (Cai et al., 2020).

The functionality and thus biomedical application of 3D scaffolds is highly correlated with their pore size and porosity. As a proven fact, an open porous structure with interconnected pores is crucial to support cell migration, proliferation, and nutrient/waste exchange, and indirectly promotes tissue regeneration and tissue vascularization (Loh and Choong, 2013). Moreover, interconnectivity of pores is determining in new tissue formation and guiding (Lutzweiler et al., 2020). The BET results including average pore diameter, total pore volume, and surface area are tabulated in Table 2. According to these data the DTL scaffold is mesoporous (with an average pore size of >2 nm and ≤50 nm) and is moderately porous, thus can be regarded as a proper candidate for tissue engineering applications (Salehi et al., 2020a; Esmaeili et al., 2022).

**TABLE 2:** The porosity characteristics of the DTL scaffold measured through BET analysis.

Scaffold	Surface area a_s, BET_ (m^2^ g^−1^)	Total pore volume (cm^3^ g^−1^)	Average pore diameter (nm)
Decellularized tomato leaf	8.25	0.013	6

A proper level of swelling in a scaffold assures efficient transfer of nutrients and metabolites within its internal area for cells and optimum assimilation of fluids. As measured in this study, the DTL scaffold was swollen up to 1365% after 24 h (Figure 2G). Thus, the scaffold shows a favorable surface/volume ratio and a sufficiently high-water holding capacity, which are beneficial for cellular liver cancer biology and pharmaceutical studies. An appropriate porosity level enables a material to interact with the fluids available in the environment and to hold enough biological fluids within the scaffold’s microarchitecture. As a result, the scaffold can function similar to a natural tissue, maintain physiological stability, and allow for the transfer of essential biomolecules and drugs (Mahendiran et al., 2021). Despite the different merits of a highly porous structure, a largely exposed surface area could lead to extensive degradation of the scaffold in a short time. Therefore, monitoring the degradation kinetics of a porous scaffold is crucial due to its direct impact on cell viability, proliferation, and host response during the tissue repairing process. The degradation rate of the DTL scaffold was tracked for 90 days. As shown in Figure 2H, the scaffold is quite stable for up to 20 days and thereafter it is completely degraded until the 90th day. This behavior could be ascribed to the low degradation rate of cellulose (Cai et al., 2020).

### 3.2 Biological characteristics of the DTL scaffold

#### 3.2.1 Cytotoxicity, cell viability, and cell morphology

**FIGURE 3:**
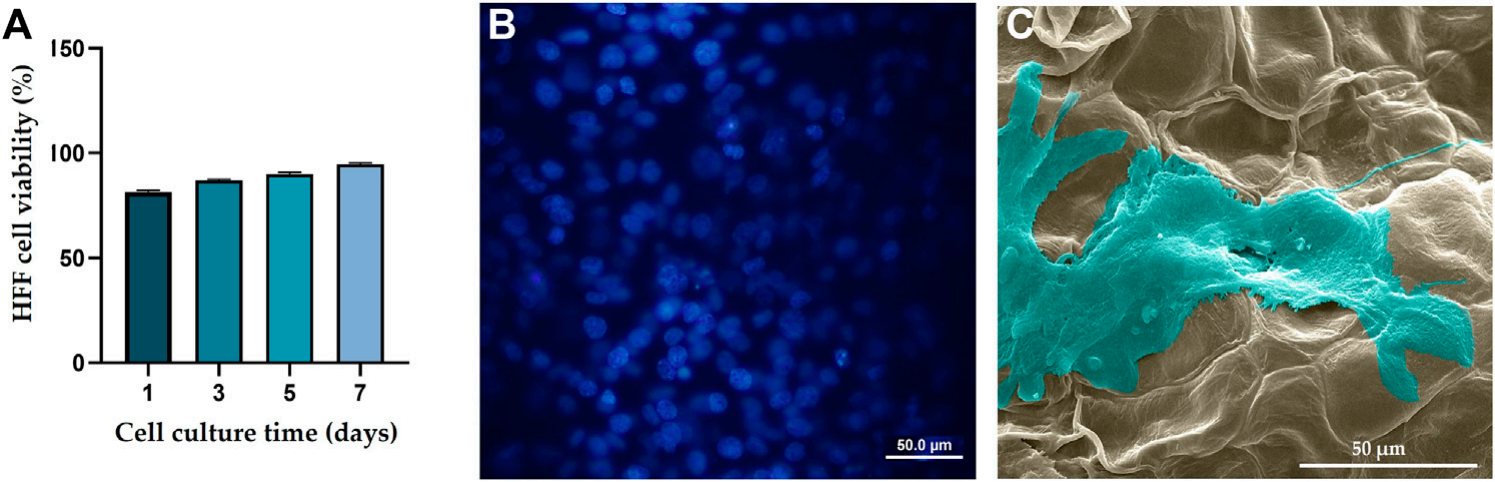
**(A)** HFF cell viability on the DTL scaffold over a 7-day incubation period. **(B)** DAPI staining image shows the live cell population seeded on the DTL scaffold. **(C)** SEM image of the HFF cells adhered and flattened on the DTL scaffold surface after 7 days.

According to a cell viability test (Figure 3A), over a 7-day culture period, viability of the HFF cells cultured on the DTL scaffold increased. The nontoxicity of the scaffold is clearly reflected in the DAPI staining and the SEM images, where the cells have proliferated and expanded on the surface of the DTL scaffold (Figures 3B, C).

**FIGURE 4:**
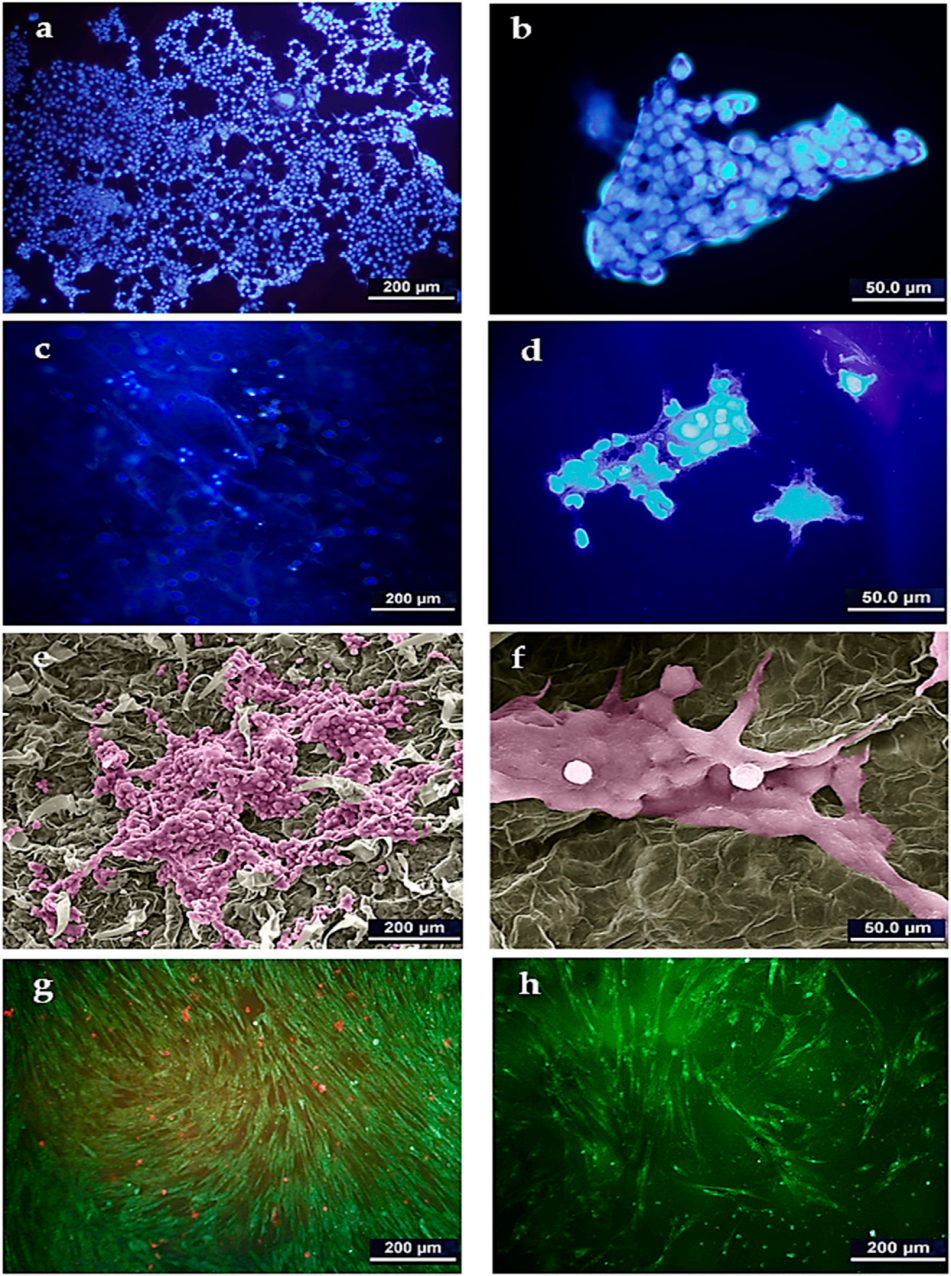
DAPI staining image of HepG2 cells on TCP control **(A)**: 500x and **(B)** 2000x) and on the DTL scaffold **(C)**: 500x and **(D)** 2000x). SEM image of HepG2 cells on the DTL scaffold after 14 days incubation **(E)**: 500x and **(F)** 2000x). The cells were colored by using Adobe photoshop 2022. AO/PI staining images of HepG2 cells cultured on: **(G)** TCP and, **(H)** DTL scaffold after 5 days incubation.

The supportive or preventive role of the DTL scaffold for HepG2 cells was identified through DAPI staining analysis and SEM imaging. As clearly seen in Figures 4A–D, HepG2 cell colonies form on the scaffold surface, as similarly do on the control (TCP) surface. Figures 4E, F show the morphology of the cells adhered onto the DTL scaffold. Like HFF cells, HepG2 cells properly interact with the scaffold surface and largely expand on it. These outcomes verify the potential of the scaffold surface in promotion of adherence, growth, and proliferation of cancerous and non-cancerous cells on the scaffold.

The AO/PI staining assay (Figures 4G, H) revealed that, despite the faster growth of HepG2 cells on the 2D TCP, a larger number of dead cells is available on TCP as compared to that on the 3D DTL scaffold. This behavior implies the improved cell survival and proliferation on the 3D scaffold.

#### 3.2.2 Clonogenic assay

**FIGURE 5:**
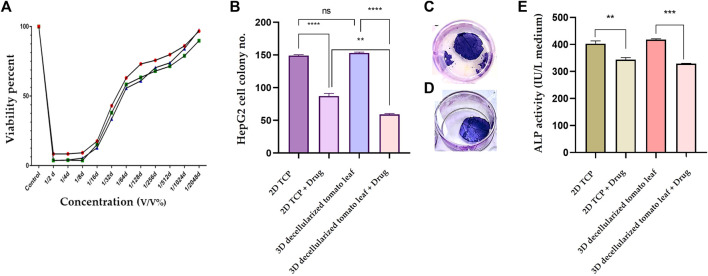
**(A)** Normal cell viability percentage after treatment with different concentrations of prilocaine for 72 h. **(B)** The number of the HepG2 cell colonies formed on the 2D TCP and the 3D DTL scaffold before and after the drug treatment (ns: non-significant, **: *p* < 0.01, and ****: *p* < 0.0001). Crystal violet staining of cells on the DTL scaffold before **(C)** and after **(D)** the drug treatment. **(E)** Measurement of ALP activity of the HepG2 cells exposed to the 2D TCP and the 3D DTL scaffold before and after the drug treatment (**: *p* < 0.01, and ***: *p* < 0.001).

To determine the most optimum prilocaine concentration, a MTT assay was performed and 1/32 (3.6 mM) concentration was considered as the half maximal inhibitory concentration (IC_50_) of the drug (Figure 5A). The number of cell colonies on the 2D substrate (TCP) and the 3D DTL scaffold before and after drug (prilocaine) treatment was quantified. As seen in Figure 5B, before the drug treatment, the number of HepG2 cell colonies on the 3D scaffold is comparable with that on TCP. After the drug treatment, regardless of the type of substrate/scaffold, the number of cell colonies decreases, implying the effectiveness of the drug in inhibiting the cancer cells (Figure 5C, D). The drug induced reduction in the number of cell colonies was more significant on the 3D DTL scaffold. This shows that the DTL scaffold can enhance the cells' response to the drug.

#### 3.2.3 ALP activity

ALP is an indicator for liver function and its upregulation might be related to biliary tract disease (Ruoß et al., 2020)**.** Therefore, ALP activity can be reliably employed in anticipation of hepatocellular carcinoma (Huang et al., 2022). We measured the ALP activity of HepG2 cells before and after the drug treatment on the 3D DTL scaffold and on the 2D TCP. As shown in Figure 5E, the ALP activity of the HepG2 cells seeded on the 3D scaffold slightly prevails over that on the 2D TCP. On the other hand, the drug treatment significantly decreases the ALP activity of the cells on both 2D and 3D substrates.

#### 3.2.4 Real-Time PCR

**FIGURE 6:**
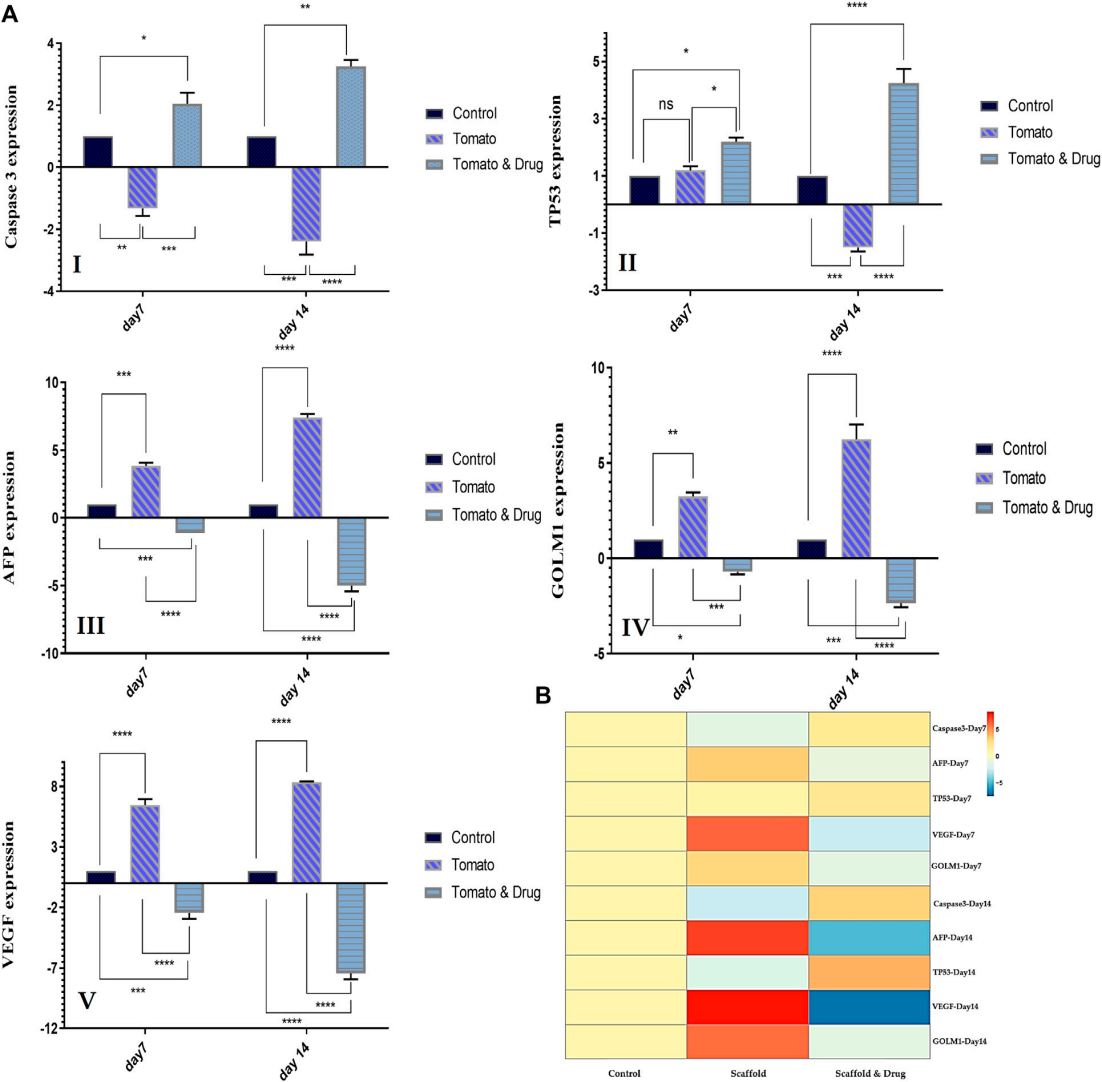
**(A)** The relative expression level of HCC-related genes (Caspase3 I, TP53 (II), AFP (III), GOLM1 (IV), and VEGF(V)) for the HepG2 cells cultured on TCP (control) and on the DTL scaffold before and after the drug treatment on the 7th and 14th days of cell culture (ns: non-significant, *: *p* < 0.05, **: *p* < 0.01, ***: *p* < 0.001, and ****: *p* < 0.0001). **(B)** Genes expression heat map of the HepG2 cells co-cultured with the 3D DTL scaffold before and after the drug treatment.

Figures 6A, B shows the gene expression level of the liver cancer (HepG2) cells seeded on the DTL scaffold and exposed to the drug, quantified via RT-qPCR. According to these results, expression of caspase 3 and TP53 genes decreased after 7 and 14 days seeding of the cells on the scaffold, while the expression of AFP, GOLM1, and VEGF genes increased. These changes indicate the growth and proliferation of liver cancer cells. On the other hand, exposure of the cells to prilocaine led to increased expression level of caspase 3 and TP53 genes and decreased expression level of AFP, GOLM1, and VEGF genes, implying the cell damage and the effectiveness of the drug in inhibiting cancer cells.

## 4 Discussion

The biochemical, mechanical, and architectural characteristics of extracellular matrix (ECM) are crucial in cancer development (Ruoß et al., 2020). For this reason, cancer biologists have developed novel technologies for *in vitro* tumor modelling using the ECM mimicking culture systems (Gill and West, 2014). *In vitro* cancer models range from simple 2D monolayers to complex 3D models that recapitulate the tumor microenvironment (Katt et al., 2016). The 2D cultured cells attach to a rigid surface and adopt a flat appearance, thus their normal activities such as signaling, proliferation, migration, and apoptosis are largely affected (Saydé et al., 2021). Instead, 3D models are novel strategies that realize *in vitro* cancer conditions and thus have attracted attentions for tissue engineering and state of the art drug screening (Chiew et al., 2017). Until now, 3D models of HCC, including HCC co-cultures, spheroids or tumoroids, organoids, bio-printed and 3D-printed models, and HCC-on-chip, have been developed. However, many of them are technically challenging and expensive and require proper facilities (Takai et al., 2016; Fasolino et al., 2017; Sontheimer-Phelps et al., 2019; Štampar et al., 2019; Taymour et al., 2021).

Scaffolding is another valuable 3D culture method (Blidisel et al., 2021). Scaffolds are prefabricated 3D constructs that are manipulated to recreate the microenvironment or ECM of particular tissues and tumors (Gao and Callanan, 2021). They are composed of diverse natural or synthetic materials with different stiffness, porosity, permeability, and surface properties (Ruoß et al., 2020).

Unlike the scaffold-free models, cells infiltrate into a 3D scaffold and therefore, physicochemical features of the scaffold material have a significant effect on cell activities (Langhans, 2018). When choosing 3D scaffolds for cell culture, a couple of parameters should be considered, including material and structural properties (such as mechanical properties, degradation, and swelling capacity), degradation byproducts with likely immunogenic reactions, and mimicry to human tumor microenvironment (Hoarau-Véchot et al., 2018).

Decellularized materials are becoming more and more attractive due to their natural structure, which is challenging for synthetic materials to recreate. They also have relatively high bioactivity, low immunogenicity, and acceptable biodegradability (Liao et al., 2020). In contrast to allografts or autografts derived from limited sources (i.e., animal and human donors), decellularized plant tissues are easily accessible and cost-effective alternatives (Modulevsky et al., 2016).

In this research, we used tomato leaves that were chemically decellularized to remove any cytotoxic substances and to improve their biocompatibility. The DNA content of the scaffold was then determined to verify the success of the decellularization process.

The FTIR analysis confirmed that the major component of the decellularized tomato leaves is cellulose. Plants are indeed suitable sources for the development of biomaterials, considering their similar mechanical and physical characteristics with biological tissues. Any plant species is unique with widely different leaf hardness and strength, which are principally caused by cell walls that are made of enduring, cellulosic (fiber-rich) composites (Wang et al., 2010; Patole et al., 2015). As a proven fact, it is possible to replicate the specific stiffness of a particular tissue, its health condition, or even disease progression using the cellulose scaffolds made from decellularized plants (Hickey and Pelling, 2019). For instance, tumor and fibrotic tissues are both known to be stiffer than normal tissues (Lacombe et al., 2020); therefore, leaf scaffolds may be chosen to represent either a tumor or a normal microenvironment based on their stiffness. The current study demonstrated that HepG2 cells can properly attach, grow, and form colony on the DTL scaffolds, thanks to promising biomimicry of tomato leaves with microscopic surface trichomes to liver cancer cells' microenvironment. Decellularized tomato leaves can be treated quickly and easily because they do not require advanced instruments or complex preparatory procedures. On the contrary, the development of synthetic scaffolds needs further time and equipment. For example, creating a 3D model of HCC based on a synthetic scaffold made of HA/poly (methylvinylether-alt-maleic acid) (HA3P50) or based on polycaprolactone (PCL) electrospun scaffolds, involves a number of challenging procedures that need specific materials and sophisticated instruments (Gao and Callanan, 2021; Turtoi et al., 2021).

An important factor that can tailor cell adhesion and cell proliferation on a scaffold is surface hydrophilicity. We treated the scaffolds with plasma to improve their hydrophilicity. As a result, the scaffolds largely absorbed PBS during a swelling test and maintained their saturation level for 24 h. The degradation analysis of the scaffolds showed their proper stability during the cell culture period.

In traditional 2D cultures, hepatocytes quickly lose their metabolic abilities because of their inadequate interaction with the surrounding matrix (Bachmann et al., 2015). Whereas, 3D culture techniques can support the maintenance of cellular metabolism over an extended period of time (Ruoß et al., 2018). The cell seeding on a 3D scaffold guarantees uniform cell adhesion not only on the plane of the cell culture plate, but rather in three dimensions, which sustains metabolic activity. Additionally, with a porous surface, the scaffold can properly supply cell nutrients (Ruoß et al., 2020). The BET results confirmed the presence of pores throughout the DTL scaffold with an average diameter of 6 nm.

Aligned with the MTT results, the DAPI staining images showed promising viability of normal cells on the DTL scaffold. This outcome proves that the scaffold is safe for cell culture and does not release any cytotoxic byproducts. Additionally, SEM and DAPI images demonstrated the formation of HCC colonies, and AO/PI images confirmed the increase in cell viability in the same period compared to a 2D substrate (control). An increase in serum ALP levels is frequently associated with a variety of diseases, such as extrahepatic bile obstruction, intrahepatic cholestasis, infiltrative liver disease, and hepatitis (Saif et al., 2005). In the current study, the increased ALP activity of the HepG2 cells seeded on the DTL scaffold implied their optimum activity.

To demonstrate the applicability of the 3D DTL scaffold in drug screening and to investigate the effect of a new drug in inhibiting liver cancer cells, we used a local anesthetic drug. Previously, inhibitory effect of local anesthetic lidocaine on the proliferation of HepG2 cells was confirmed (Xing et al., 2017). In this study, we used the local anesthetic drug of prilocaine and its effects on the cancer cells were monitored through a clonogenic assay, and ALP activity and gene expression measurement. The clonogenic assay showed a decrease in the number of cancer cell colonies and in ALP activity after the drug treatment. This outcome implies the effectiveness of the drug (coupled with the 3D DTL scaffold) in inhibiting cancer cells. We noticed that seeding HepG2 cells on the 3D scaffold promotes their response to prilocaine. Such feedback shows that the tumor niche modulates the cytotoxic response to a particular drug (Henke et al., 2020).

The expression of HCC-related genes was investigated to confirm the growth of cancer cells on the 3D scaffold and the effectiveness of the drug. The AFP gene produces alpha-fetoprotein, which is a dominant plasma protein generated by liver and yolk sac over the course of fetal life. The expression of alpha-fetoprotein in adults could indicate hepatocarcinoma (Chen et al., 2020). VEGF is the main regulator of angiogenesis during growth and development, as well as in disease states such as cancer, and is an important mediator in vascular growth in tumors. VEGF can possibly contribute to HCC invasion and metastasis. Tumor angiogenesis is associated with HCC progression (Oliveira et al., 2020). GOLM1, a Golgi membrane protein, is commonly produced in epithelial cells and its expression is upregulated under the influence of viral infection. GOLM1 can induce HCC development and metastasis and can be an important marker for the early detection of HCC (Yan et al., 2020). According to RT-PCR results, the expression of AFP, VEGF, and GOLM1 genes increased from day 7 to day 14 of culturing cells on the DTL scaffold. The expression of these three genes decreased from day 7 to day 14 as a result of the drug treatment. Caspase-3, a downstream effector cysteine protease in the apoptotic pathway, is commonly expressed in various human tissues (e.g., liver) and its activation is associated to cell damage and apoptosis (Deng et al., 2022). TP53 is recognized for its function as a tumor suppressor. It detects cellular stress or damage and, in response, prevents cell division or initiates cell death, thereby preventing the proliferation of damaged cells. The mutation of this gene destroys a key cellular mechanism and leads to cancer (Long et al., 2019). The expression patterns of Caspase 3 and TP53 in the cells exposed to the 3D scaffold decreased from day 7 to day 14, in comparison to those on the 2D substrate (control). However, the drug treatment provoked the expression level of these two genes. Therefore, HepG2 cells were shown to grow adequately on the 3D scaffold and prilocaine could inhibit the growth of cancer cells effectively.

Cancer cell lines are frequently used for developing and for characterizing new cellular models and for investigating new treatment strategies. Here, we chose the HepG2 cell line, which is widely used for *in vitro* modelling of cancer and drug screening studies. Since plant derived models have been appealing in construction of tissue engineering scaffolds, it is necessary to examine how normal and cancer cells respond to such scaffolds (Iravani and Varma, 2019).

The purpose of the current study was to investigate HepG2 cell adhesion, survival rate, and proliferation on a decellularized plant-based scaffold. Decellularized tomato leaves with their unique surface morphology and proper physicochemical properties, provided a suitable substrate for the growth and colony formation of liver cancer cells. According to the data reported here, this cellulose-based scaffold can resemble the HCC microenvironment and be applied reliably for 3D modelling of liver cancer. With respect to decellularized plant derived scaffolds, there are still many aspects that require further investigation. For instance, the lack of optimized decellularization protocols and necessary characterization approaches with respect to mechanical and *in vivo* biological properties should be circumvented. In this regard, the interplay between mechanical properties and topography of decellularized plant scaffolds and cell behavior and cell organization should be validated. Moreover, biofunctionalization, biodegradability, heterogeneity, and reproducibility of decellularized plant based scaffolds need to be studied (Harris et al., 2021). Taken together, plant species with different morphology and stiffness, can host a wide range of cells and be used as promising scaffolds in tissue engineering.

## 5 Conclusion

In the current study, an *in vitro* cellulose-based 3D model of HCC was developed, and its cell behavior was compared with that of a conventional 2D substrate. Tomato leaves after decellularization can be used as affordable, biocompatible, and non-toxic scaffolds for *in vitro* 3D cancer modeling. Our investigations, including SEM imaging, cell attachment and viability assays, and gene expression analysis, verified that cells could grow properly on the scaffold. Additionally, culturing cells on these scaffolds improved the cell response to the drug and increased cell survival in comparison to a 2D substrate model, demonstrating efficiency of these scaffolds for drug screening. In conclusion, this cellulose scaffold can be used as a 3D platform for modelling liver cancer, drug screening, and discovery of new treatment strategies.

## Data Availability

The original contributions presented in the study are included in the article/supplementary material, further inquiries can be directed to the corresponding authors.
